# Highly Effective Removal of Ofloxacin from Water with Copper-Doped ZIF-8

**DOI:** 10.3390/molecules27134312

**Published:** 2022-07-05

**Authors:** Xiaowei Wang, Yingjie Zhao, Yiqi Sun, Dahuan Liu

**Affiliations:** 1 College of Chemical Engineering, Beijing University of Chemical Technology, Beijing 100029, China; wangxw9743@163.com (X.W.); zhaoyj0215@163.com (Y.Z.); 2 College of Materials Science and Engineering, Beijing University of Chemical Technology, Beijing 100029, China; 2019020092@mail.buct.edu.cn

**Keywords:** adsorption, complexation, metal-organic frameworks, ofloxacin

## Abstract

Residual antibiotics in wastewater have gained widespread attention because of their toxicity to humans and the environment. In this work, Cu-doped ZIF-8s (Cu-ZIF-8s) were successfully synthesized by the impregnation of Cu^2+^ in ZIF-8 and applied in the removal of ofloxacin (OFX) from water. Remarkably, excellent adsorption performance was obtained in Cu-ZIF-8s, especially for Cu-ZIF-8-1, in which the adsorption capacity (599.96 mg·g^−1^) was 4.2 times higher than that of ZIF-8 and superior to various adsorbents reported previously. The adsorption kinetics and adsorption isotherm follow the pseudo-second-order model and the Langmuir model, respectively. Furthermore, the removal efficiencies of OFX in Cu-ZIF-8-1 reached over 90% at low concentrations. It was revealed that electrostatic interaction and complexation play important roles in the adsorption process. In addition, the material can be regenerated by simple methods. Therefore, the obtained Cu-doped MOFs may have a promising application in the treatment of antibiotic-containing wastewater.

## 1. Introduction

With the continuous development of science and technology as well as the industrialization process, various kinds of drugs are widely used for the treatment and prevention of diseases [1]. However, most antibiotics are not fully metabolized in humans and cannot be biodegraded in the natural environment, which has led to a series of water pollution problems [2,3]. Antibiotics can be detected in sewage from wastewater treatment plants, surface water, and groundwater. With the increase in public awareness of environmental protection, water security has received increased attention [4]. Ofloxacin (OFX) is reported to be one of the most used antibiotics in poultry and aquaculture worldwide, and its massive usage poses a huge threat to the environment [5]. It inhibits the physiological processes of the natural photochemical and antioxidant systems of algae and reduces algal cell growth, chlorophyll content, and the photosynthetic rate. More importantly, increased OFX residues in the environment can lead to increased drug resistance. In addition, OFX can cause acute toxicity to aquatic organisms at mg·L^−1^ levels and chronic toxicity at μg·L^−1^ levels [6,7]. Therefore, it is necessary to efficiently remove OFX from the water.

Several methods have been used for the removal of OFX from water, such as biological [8], sonochemical [9], and ozonation methods [10]. Unfortunately, the disadvantages of low efficiency, high cost, and the generation of toxic by-products limit the widespread application of these methods. By contrast, adsorption is considered to be promising in water treatment. Several kinds of adsorbents have been used to adsorb OFX from water, including clay [11,12,13], activated carbon [8,14,15,16,17,18,19], carbon nanotubes [20,21,22,23], graphene oxide [24,25,26,27], and biochar [28,29,30]. However, most of these still present the problem of low adsorption capacity. Thus, there is an urgent need to develop new adsorbents for capturing this antibiotic.

Metal-organic frameworks (MOFs) are a new class of porous materials composed of inorganic metals and organic ligands. Due to their high specific surface area, high porosity, and easy chemical modifiability, they have been successfully used for the adsorptive removal of pollutants from water [31,32,33,34]. However, owing to the lack of active sites, the adsorption performances of many MOFs need to be further improved. Toward this target, an efficient strategy is to incorporate metal elements into MOFs to increase the number of active sites. Herein, the Cu element was adopted to synthesize a Cu-doped ZIF-8 using the impregnation method to remove OFX from water. Compared with that of the pristine ZIF-8, the adsorption capacities in Cu-doped ZIF-8s were increased by up to 4.2 times owing to the complexation of Cu with OFX. Specifically, Cu-ZIF-8-1 showed a high adsorption capacity of 599.96 mg·g^−1^, which is superior to various adsorbents reported previously. The adsorption behaviors, including adsorption kinetics, isotherm, the effect of pH, the effect of coexisting ions, and reusability, were also investigated. These results indicate that the obtained Cu-ZIF-8s not only have great potential to efficiently remove OFX from water but also provide a way to apply metal-doped MOF adsorbents in the adsorption of pharmaceuticals.

## 2. Experimental Section

### 2.1. Chemicals

Zinc nitrate hexahydrate (Zn(NO_3_)_2_·6H_2_O, 99.99%) was purchased from Shanghai Aladdin Reagent Co., Ltd. (Shanghai, China). Copper nitrate trihydrate (Cu(NO_3_)_2_·3H_2_O, 99%) and 2-Methylimidazole (C_4_H_6_N_2_, 99%) were purchased from Beijing J&K Scientific Co., Ltd. (Beijing, China). Methanol (CH_4_O, ≥99.5%) and anhydrous ethanol (C_2_H_6_O, ≥99.7%) were purchased from Tianjin Fuyu Fine Chemical Co., Ltd. (Tianjin, China). OFX (C_18_H_20_FN_3_O_4_, 98%) was provided by Shanghai Maclean Biochemical Technology Co., Ltd. (Shanghai, China). All chemical reagents were used without further purification.

### 2.2. Preparation of Materials

For the synthesis of ZIF-8, 5.95 g (20 mmol) of Zn(NO_3_)_2_·6H_2_O and 6.16 g of (75 mmol) 2-methylimidazole were first dissolved in 150 mL of methanol and recorded as solution A and solution B, respectively. Then, solution B was slowly added to solution A by stirring at room temperature for 24 h. The white precipitate was collected by centrifugation at 10,000 rpm for 10 min and washed with methanol at least 6 times in 2 days. Finally, ZIF-8 was obtained overnight at 60 °C under vacuum.

Cu-ZIF-8s were synthesized according to the previous method with slight modifications [35]. First, 0.5 g of ZIF-8 was added to 40 mL of ethanol solution containing 0.12 g (0.5 mmol), 0.24 g (1 mmol), or 0.36 g (1.5 mmol) of Cu(NO_3_)_2_·3H_2_O. The solution was stirred at room temperature for 3 h. The light blue precipitate was obtained by centrifugation at 10,000 rpm for 10 min and washed with ethanol at least 6 times in 2 days. Finally, the obtained Cu-ZIF-8-x was dried overnight at 60 °C under vacuum; x represents the added amounts of copper salt, which were 0.5, 1, and 1.5.

### 2.3. Characterization of Materials

The 77 K N_2_ adsorption–desorption was determined using a 3H-2000BSD-PS1/2A series of automatic surface and aperture analyzers (Beishide Instrument Technology Co., Ltd. (Beijing, China)). PXRD patterns were performed on a D8 Advance X diffractometer equipped with Cu Kα radiation (λ = 1.54178 Å) at room temperature. FT-IR data were collected using a Nicolet 6700 FT-IR spectrophotometer. X-ray photoelectron spectroscopy (XPS) data were collected by a Thermo Fisher ESCALAB (Shanghai, China). A Zetasizer Nano ZS90 zeta potential analyzer was used to measure the zeta potential data. The concentration of OFX was analyzed by a TU-1901 UV–vis spectrophotometer (Purkinje General Instrument Co., Ltd., Beijing, China). Scanning electron microscopy (SEM, TESCAN MIRA LMS) was applied to characterize the morphology, and energy-dispersive X-ray spectroscopy (EDS, Xplore) elemental mapping spectrum of materials was obtained using SEM analysis. An inductively coupled plasma optical emission spectrometer (ICP–OES) (Thermo Fisher iCAP PRO (OES) (Shanghai, China)) was used to measure the metal contents in the samples.

### 2.4. Experiments of Adsorption

All adsorption experiments in this work were performed in 20 mL glass vials at 303 K. During the adsorption process, except for the adsorption isotherm, the other experiments were conducted by adding 10 mg of adsorbents into 10 mL of aqueous solution of OFX. These glass vials were then placed in a thermostatic shaking oscillator at 150 rpm for a predetermined time. After the adsorption experiment, the suspension was filtered through a polyethersulfone microporous filter membrane with a 0.22 μm pore size (Beijing Zhuoxin Hongye Instruments & Equipment Co., Ltd. (Beijing, China)), and the concentration of OFX of the collected clarified filtrate was determined. To evaluate the effect of pH, the pH value of the OFX solution was adjusted using 0.1 M NaOH and 0.1 M HCl. The amount of adsorbed OFX (*q*_e_ mg·g^−1^) was calculated using the following equation:

$$q_{e}=\frac{\left(c_{0}-c_{e}\right)\times V}{m}$$

where *q*_e_ (mg·g^−1^) is the adsorption amount; *c*_0_ (mg·L^−1^) and *c*_e_ (mg·L^−1^) are the initial and equilibrium concentrations of OFX, respectively; *V* (L) is the volume of the solution; and *m* (g) is the mass of the adsorbent.

## 3. Results and Discussion

### 3.1. Characterization of MOFs

**Figure 1:**
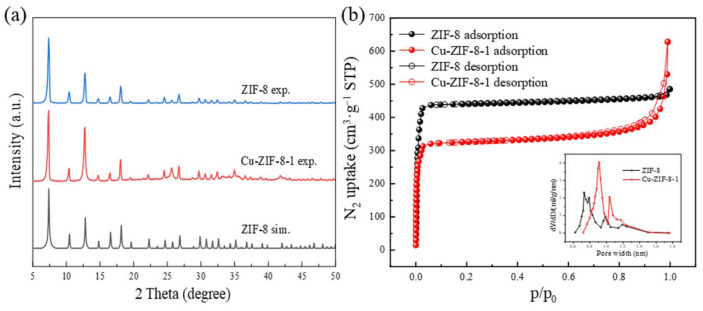
Powder X-ray diffraction (PXRD) patterns (**a**); N_2_ adsorption-desorption isotherms and pore size distribution (**b**) of ZIF-8 and Cu-ZIF-8-1.

The crystal structures of ZIF-8 and Cu-ZIF-8s were analyzed by PXRD, and the results are shown in Figure 1a using Cu-ZIF-8-1 as an example. ZIF-8 had eight major characteristic peaks at 2θ of 7.3, 10.4, 12.7, 14.7, 16.4, 18.0, 24.5 and 26.7°, which matched well with the simulated ones, indicating successful synthesis. When Cu was doped into ZIF-8, the diffraction peaks in the PXRD patterns were almost the same as those of ZIF-8, as shown in Figure 1a. No diffraction peaks of Cu species could be observed, indicating that the addition of Cu did not change the basic structure of ZIF-8, and the Cu element was probably incorporated into the framework (Figure 1a and Figure S1). Moreover, the PXRD patterns of Cu-ZIF-8-1 remained almost unchanged even after immersion in water for 15 days, which indicates that Cu-ZIF-8-1 has good water stability (Figure S2).

To investigate the effect of Cu-doping on the pores, N_2_ adsorption–desorption isotherms at 77 K were conducted to obtain a specific surface area and porosity [36,37]. As shown in Figure 1b and Figure S3 and Table 1 and Table S1, the BET specific surface area slightly decreased with an increase in the Cu content, and it was significantly lower than that of the pristine ZIF-8. This may be due to the fact that during the formation of Cu-ZIF-8s, Cu ions may replace some of the Zn ions in the framework, and the addition of these ions may damage some of the linkers [35]. The large pore size of Cu-ZIF-8s is conducive to the accessibility of drug molecules, and the doped Cu element may provide additional adsorption sites; both these factors are beneficial for increasing the adsorption capacity. In addition, the contents of Cu and Zn in Cu-ZIF-8s were measured by ICP–MS characterization. The results are shown in Table S2. With increased Cu loading, the Cu:Zn ratios were 1:6.79, 1:3.01, and 1:1.74 in the obtained samples, respectively. It can be seen that the content of Zn was decreased, indicating that Cu replaced part of Zn in the framework.

The FT-IR spectra of ZIF-8 and Cu-ZIF-8-1 are shown in Figure S4. The main peak at 3448.6 cm^−1^ was mainly due to the -OH stretching vibration caused by the adsorbed water molecules. The peak at 2928.9 cm^−1^ was attributed to the aromatic and the aliphatic C-H stretching of the imidazole [38]. The characteristic peak at 3137.1 cm^−1^ was mainly due to C-N stretching on the imidazole ring, while the peak at 1581.9 cm^−1^ corresponded to the stretching vibration of C = N [39]. Obviously, these characteristic peaks still exist in the FT-IR spectrum of Cu-ZIF-8-1, indicating that the Cu-doped modification did not change the structure of ZIF-8, as revealed by the results of PXRD.

**Figure 2:**
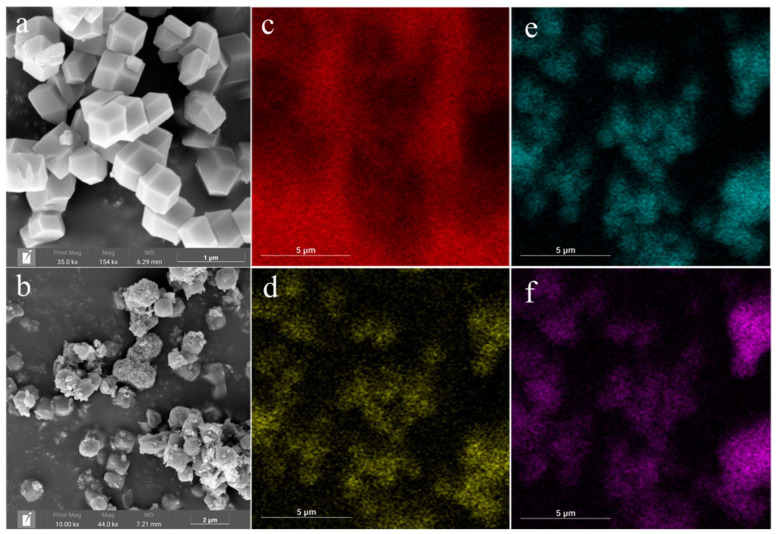
SEM images of ZIF-8 (**a**) and Cu-ZIF-8-1 (**b**); EDS mappings of the selected areas in Cu-ZIF-8-1: distribution of C (**c**), N (**d**), Zn (**e**), and Cu (**f**).

To visualize the effect of Cu-doping on ZIF-8, the morphologies were obtained by SEM. As shown in Figure 2a, ZIF-8 had a regular rhombic dodecahedral morphology with a smooth, flat surface and a particle size of about 500 nm. After Cu-doping, the particle size and morphology were similar to those of the pristine ZIF-8 (Figure 2b). However, the surface became obviously rough, which indicates that the doping process destroys part of the ligand and replaces part of Zn in the framework [35]. In addition, the EDS elemental mapping spectrum of Cu-ZIF-8-1 was also obtained using SEM. As shown in the mapping of C, N, Zn, and Cu elements in Figure 2c–f, Cu was uniformly distributed in Cu-ZIF-8-1.

### 3.2. Study of Adsorption

#### 3.2.1. Effect of Cu Loading

**Figure 3:**
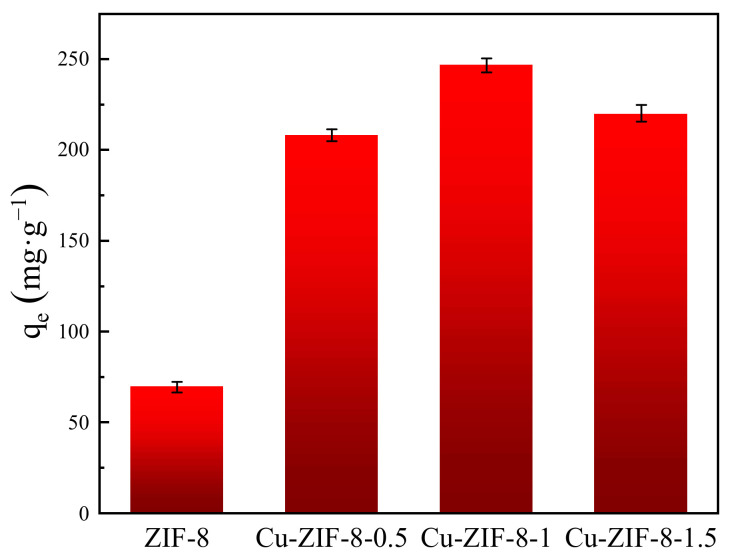
Adsorption capacity at different loadings (*c*_0_ = 270 mg·L^−1^).

The effect of different concentrations of Cu^2+^ on adsorption was investigated in the first step to determine the optimal loading. As shown in Figure 3, the adsorption capacity of Cu-ZIF-8s was larger than that of ZIF-8. Notably, Cu-ZIF-8-1 exhibited a higher adsorption capacity than others. This is because more Cu loadings provided more active sites that can interact with OFX, resulting in a higher adsorption capacity. However, the excess loadings may lead to severe breaks in organic linkers, resulting in low specific surface areas and corresponding low adsorption capacities [35]. Therefore, the subsequent adsorption studies were carried out with Cu-ZIF-8-1 and ZIF-8.

#### 3.2.2. Adsorption Kinetics

**Figure 4:**
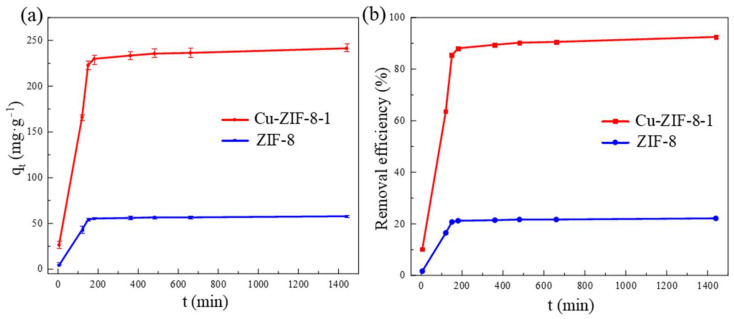
Adsorption amounts (**a**) and removal efficiency (**b**) of OFX as a function of time (*c*_0_ = 260 mg·L^−1^).

First, the adsorption amount of OFX on ZIF-8 and Cu-ZIF-8-1 was investigated as a function of time with the condition of an initial concentration of 260 mg·L^−1^. In Figure 4, it can be seen that the adsorption equilibrium can be reached at 180 min, which is favorable for the removal of pollutants. The equilibrium adsorption capacity of Cu-ZIF-8-1 reached 241.43 mg·g^−1^, which was 4.17 times higher than that of ZIF-8. The removal rate of Cu-ZIF-8-1 over OFX was 92%.

The adsorption kinetics of OFX on ZIF-8 and Cu-ZIF-8-1 were further investigated using the pseudo-first-order model and the pseudo-second-order model:

Pseudo-first-order model:

$$ln(q_{e}-q_{t})=\ln q_{e}-k_{1}t$$

Pseudo-second-order model:

$$\frac{t}{q_{t}}=\frac{1}{k_{2}q_{e}{}^{2}}+\frac{t}{q_{e}}$$

where *q*_e_ (mg·g^−1^) and *q*_t_ (mg·g^−1^) are the adsorption amounts of ofloxacin at equilibrium and a certain time *t*, respectively; *k*_1_ (min^−1^) and *k*_2_ (g·mg^−1^·min^−1^) are the rate constants of the pseudo-first-order model and the pseudo-second-order model, respectively.

The fitting results are shown in Figures S5 and S6 and Table 2 and Table S2. From the comparison of the correlation parameters, it was concluded that OFX adsorption in ZIF-8 and Cu-ZIF-8-1 can be well described by the pseudo-secondary model, indicating that the adsorption process is mainly dominated by chemisorption.

#### 3.2.3. Adsorption Isotherms

**Figure 5:**
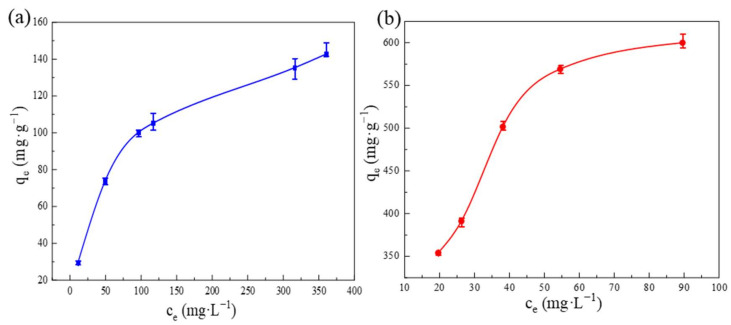
OFX adsorption isotherm on ZIF-8 (**a**) and Cu-ZIF-8-1 (**b**) (*m* = 5 mg; *V* = 10 mL).

To investigate the maximum adsorption capacity of samples for OFX as thoroughly as possible, batch experiments were conducted at different initial concentrations (120 mg·L^−1^–500 mg·L^−1^) to collect the adsorption isotherms. As shown in Figure 5, the adsorption capacity increased with the increase in OFX concentration. The maximum adsorption capacity reached 599.96 mg·g^−1^, which was 4.2 times that of ZIF-8 (142.74 mg·g^−1^). More importantly, the adsorption capacity of Cu-ZIF-8-1 was higher than that in most of the reported materials, as shown in Table S4 [11,16,40,41,42,43,44,45,46,47,48,49,50,51,52,53,54,55]. To further study the adsorption behavior, the Langmuir model and Freundlich model were used to fit the adsorption isotherms:

Langmuir isotherm model:

$$\frac{c_{e}}{q_{e}}=\frac{1}{K_{L}q_{m}}+\frac{q_{e}}{q_{m}}$$

Freundlich isotherm model:

$$lnq_{e}=\ln K_{F}+\frac{1}{n}\ln c_{e}$$

where *q*_e_ (mg·g^−1^) is the equilibrium adsorption amount, *c*_e_ (mg·L^−1^) is the equilibrium solute concentration, *q*_m_ (mg·g^−1^) is the maximum adsorption amount of the adsorbent, and *K_L_* (L·mg^−1^) is the Langmuir adsorption constant. The fitting results are shown in Figures S7 and S8 and Table 3 and Table S5. Obviously, the Langmuir model is more suitable to describe the adsorption process with homogeneous monolayer adsorption.

#### 3.2.4. Effect of Coexisting Ions

**Figure 6:**
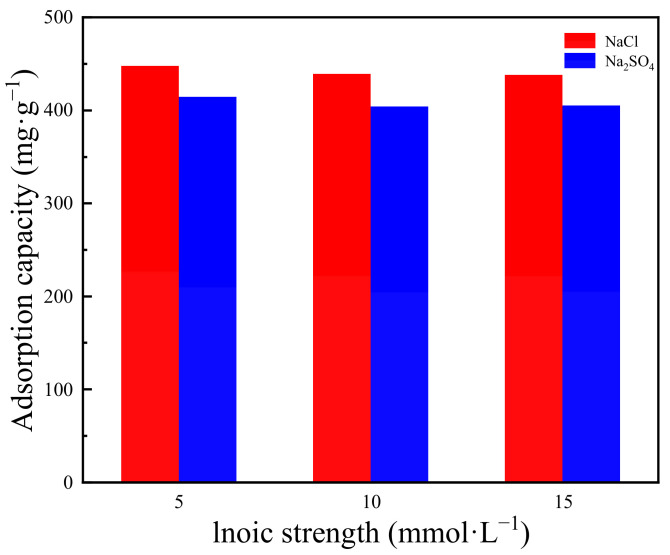
The adsorption capacity of Cu-ZIF-8-1 with the different coexisting ions (Cl^−^, SO_4_^−^) (*c*_0_ = 522 mg·L^−1^).

Inorganic ions, such as Cl^−^ and SO_4_^2−^, may be present in wastewater. Therefore, the effect of coexisting ions on the adsorption of OFX by Cu-ZIF-8-1 was also investigated in this work. As shown in Figure 6, there was a slight decrease in the adsorption amount of OFX due to the competitive adsorption with the increase in inorganic ion concentrations. From the above results, it was suggested that Cu-ZIF-8-1 may have a good anti-interference ability and thus is promising for the practical treatment of wastewater.

#### 3.2.5. Regeneration of Adsorbent

**Figure 7:**
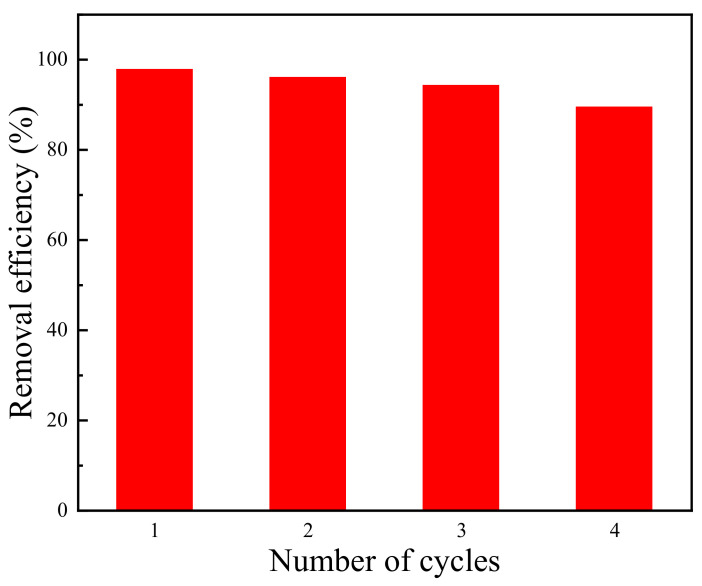
Regeneration of Cu-ZIF-8-1 for OFX adsorption (*c*_0_ = 45 mg·L^−1^).

In the practical application, the reusability of the adsorbent is important. In this work, ethanol was chosen as the eluent to remove OFX from the sample. The adsorbed Cu-ZIF-8-1 was immersed in the anhydrous ethanol solution and washed with ethanol at least 6 times in 2 days. Finally, the samples were dried under vacuum at 80 °C overnight. As shown in Figure 7, after three cycles, Cu-ZIF-8-1 still had good adsorption capacity, showing good regeneration ability.

### 3.3. Mechanism of Adsorption

**Figure 8:**
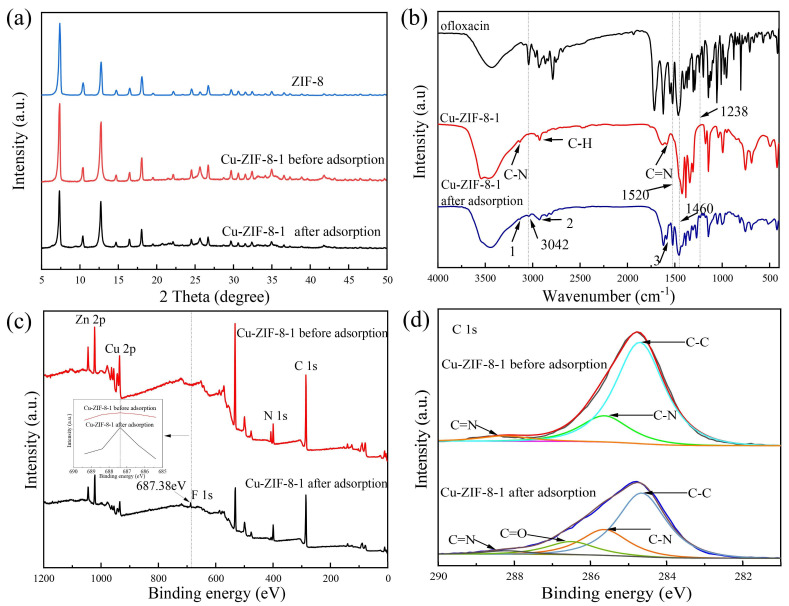
Comparison of PXRD (**a**), FT-IR spectrum (**b**), and XPS (**c**,**d**) of Cu-ZIF-8-1 before and after adsorption (*c*_0_ = 200 mg·L^−1^).

To verify whether OFX was adsorbed onto Cu-ZIF-8s, a series of characterizations were performed on the adsorbed material using Cu-ZIF-8-1 as an example. As shown in Figure 8a and Figure S9, the PXRD and SEM of the adsorbed Cu-ZIF-8-1 were similar to the original ones, indicating that the structure of Cu-ZIF-8-1 was not destroyed after adsorbing OFX. Several new peaks were found in the FT-IR spectrum after adsorption (Figure 8b). The peaks at 1460 cm^−1^, 1520 cm^−1^, 1238 cm^−1^, and 3042 cm^−1^ represent methylene (-CH_2_) in the benzoxazine ring and alkyl groups (-CH_3_ and -CH_2_), C-O-C stretching vibrations, and C-N groups in OFX, respectively [39]. Additionally, after adsorption, new peaks at 687.38 eV and 286.5 eV appeared in the XPS patterns (Figure 8c,d), which represent the F element and C=O in the OFX molecule [56]. These results confirmed that OFX was adsorbed on Cu-ZIF-8-1 and that the sample has good stability.

**Figure 9:**
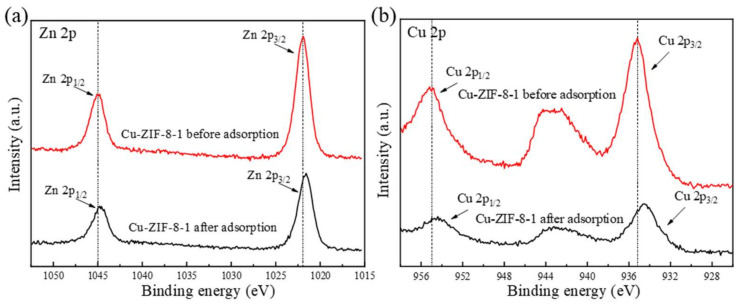
XPS spectra of Zn (**a**) and Cu (**b**) in Cu-ZIF-8-1 before and after adsorption (*c*_0_ = 200 mg·L^−1^).

Then, the adsorption mechanism of OFX in Cu-ZIF-8-1 was investigated using XPS. As shown in Figure 9a, the peaks of Zn 2p_1/2_ and Zn 2p_3/2_ were located at 1045 eV and 1022 eV [56]. The peaks at 955 eV and 935.15 eV can be attributed to Cu 2p_1/2_ and Cu 2p_3/2_ (Figure 9b) [57]. Compared with the original sample, the binding energies of Zn 2p and Cu 2p of the material after adsorbing OFX decreased by 0.3 eV and 0.75 eV, respectively, which indicates that both Zn and Cu gained electrons. The peak intensities of Zn 2p and Cu 2p became significantly weaker in the OFX-loaded sample. These demonstrate that the unsaturated metal sites in the framework may undergo complexation with -COOH in OFX during the adsorption process [39,58,59,60].

**Figure 10:**
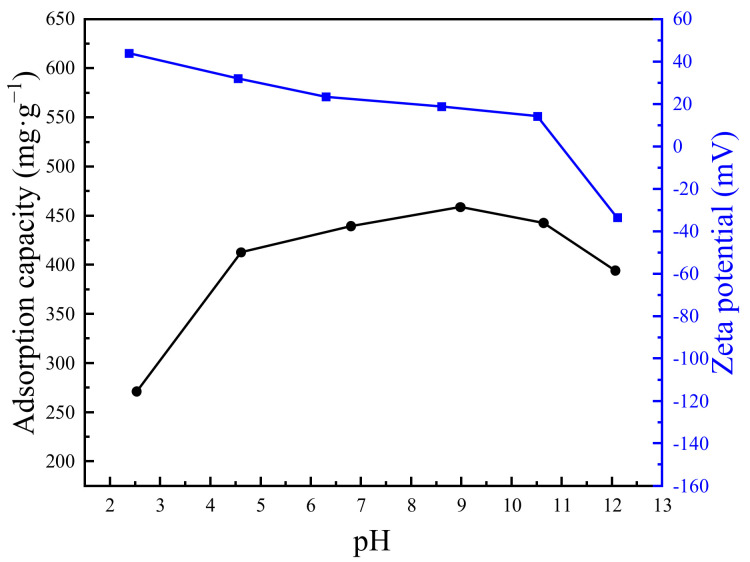
Adsorption capacity and zeta potential of Cu-ZIF-8-1 with the different pH values (*c*_0_ = 522 mg·L^−1^).

In general, the pH value of the OFX solution has a great influence on the existing state of OFX molecules, as well as the corresponding adsorption performance. Therefore, to further understand the adsorption mechanism, the effect of pH on adsorption capacity was studied. As shown in Figure 10, the surface charge of Cu-ZIF-8-1 was positive below the pH value of 11 and became negative above the pH value of 11. Meanwhile, the OFX molecules could form three species in aqueous solutions under different pH values, including cationic species (pH < 5.77), zwitter ionic species (5.77 < pH < 8.44), and anionic species (pH > 8.44) [35]. The low adsorption capacity of Cu-ZIF-8-1 at 2 < pH < 5.77 is because both Cu-ZIF-8-1 and OFX are positively charged at this range of pH and repel each other. At the pH range of 5.77–8.44, the adsorption capacity of OFX was almost unchanged since OFX molecules are neutral and electrostatic interaction has little effect on the adsorption process. For 8.44 < pH < pH_zpc_, the surface charge of the OFX molecule is negative, while Cu-ZIF-8-1 nanoparticles become positively charged, inducing the maximum adsorption capacity. For pH > pH_zpc_, the surface charge of the OFX molecule was negative, and Cu-ZIF-8-1 gradually became negatively charged, causing a decrease in the adsorption capacity [61]. Therefore, the electrostatic interaction plays an important role in the adsorption process. In combination with the discussion mentioned above, it can be concluded that the cooperative effect of the complexation and electrostatic interaction endows Cu-ZIF-8-1 with a high adsorption capacity for OFX from water. In addition, the aromatic rings of MOFs may be bound to the guest molecule via π-π interaction [62].

## 4. Conclusions

In summary, a method of impregnating supported copper ions was used to prepare Cu-ZIF-8s in this work. Remarkably, Cu-ZIF-8-1 had a large adsorption capacity for OFX, which was 4.2 times higher than that of ZIF-8 and also higher than that in most of the reported adsorbents. Compared with the PXRD and FT-IR of the adsorbent before and after adsorption, there was no significant structural damage to the adsorbent. Meanwhile, the adsorbent could be conveniently regenerated by washing with ethanol. It was observed that the complexation and electrostatic interaction played important roles in the adsorption process. In addition, π-π interaction may also promote OFX removal. The results obtained in this work indicate that Cu-ZIF-8-1 may not only serve as a potential adsorbent for OFX and even PPCPs but also provide a guideline for designing and constructing novel adsorbents with high efficiency.

## Figures and Tables

**Table 1:** Specific surface area, mesopore volume, micropore volume, and pore size of ZIF-8 and Cu-ZIF-8-1.

MOFs	S_Langmuir_ (m^2^·g^−1^)	V_t_ (m^3^·g^−1^)	D (nm)
**ZIF-8**	1929.80	0.7317	1.3515
**Cu-ZIF-8-1**	1438.27	0.7353	1.6124

**Table 2:** Kinetics models parameters of OFX adsorbed on Cu-ZIF-8-1.

MOF	Pseudo-First-Order Model			Pseudo-Second-Order Model		
	*q*_e,cal_ (mg·g^−1^)	*k*_1_ (min^−1^)	R^2^	*q*_e,cal_ (mg·g^−1^)	*k*_2_ (g·min^−1^·mg^−1^)	R^2^
Cu-ZIF-8-1	153.1611	0.0083	0.9199	247.5248	0.0001	0.9989

**Table 3:** Isotherm model parameters for OFX adsorbed on the Cu-ZIF-8-1.

MOF	Langmuir Isotherm Model			Freundlich Isotherm Model		
	*q*_m_ (mg·g^−1^)	*K*_L_ (L·g^−1^)	R^2^	K_F_ ((L·mg^−1^)1/*n* mg·g^−1^)	1/*n* (g·min^−1^·mg^−1^)	R^2^
Cu-ZIF-8-1	757.5758	0.0461	0.9872	120.8693	0.3714	0.8996

## Supplementary Materials

The following supporting information can be downloaded at: https://www.mdpi.com/article/10.3390/molecules27134312/s1, Figure S1: PXRD patterns of ZIF-8, Cu-ZIF-8-0.5, Cu-ZIF-8-1 and Cu-ZIF-8-1.5; Figure S2: PXRD pattern of Cu-ZIF-8-1 after soaking in water for 15 days; Figure S3: N2 adsorption-desorption isotherms of Cu-ZIF-8-0.5, Cu-ZIF-8-1 and Cu-ZIF-8-1.5; Table S1: Specific surface area, mesopore volume, micropore volume and pore size of samples; Table S2: Contents of Cu and Zn in Cu-ZIF-8s; Figure S4: FT-IR spectrum of ZIF-8 and Cu-ZIF-8-1; Figure S5: Fitting results of kinetic models on ZIF-8: Pseudo-first-order model (a); Pseudo-second-order model (b); Figure S6: Fitting results of kinetic models on Cu-ZIF-8-1: Pseudo-first-order model (a); Pseudo-second-order model (b); Table S3: Kinetics model parameters of OFX adsorbed on ZIF-8; Table S4: Adsorption capacities for the removal of OFX by different adsorbents; Figure S7: Fitting results of adsorption isotherms on ZIF-8: Langmuir model (a); Freundlich model (b); Figure S8: Fitting results of adsorption isotherms on Cu-ZIF-8-1: Langmuir model (a); Freundlich model (b); Table S5: Model parameters for OFX adsorbed on the ZIF-8; Figure S9: SEM images of Cu-ZIF-8-1 after adsorption (c_0_ = 200 mg·L^−1^). PXRD patterns, N_2_ adsorption–desorption isotherms, contents of Cu and Zn and FT-IR spectrum of Cu-ZIF-8s, and contents of Cu and Zn in Cu-ZIF-8s; Fitting results of the kinetic model; Adsorption capacities of OFX by different adsorbents; Fitting results of adsorption isotherms; SEM images of Cu-ZIF-8-1 after adsorption.

## Sample Availability

Samples are available from the corresponding author.
