# Supplementary material for: Highly Effective Removal of Ofloxacin from Water with Copper-Doped ZIF-8
Source: Molecules. 2022 Jul 5;27(13):4312. doi: 10.3390/molecules27134312 (PMC9268637; doi:10.3390/molecules27134312)
Supplement: Supplementary file 1 [file molecules-27-04312-s001.zip › molecules-1787039 Supplementary.pdf]

Supporting Information

Highly Effective Removal of Ofloxacin from Water with Copper-Doped ZIF-8

Xiaowei Wang[1], Yingjie Zhao[1], Yiqi Sun[2], Dahuan Liu[1]*

[1] College of Chemical Engineering, Beijing University of Chemical Technology, Beijing 100029, PR China

[2] College of Materials Science and Engineering, Beijing University of Chemical Technology, Beijing 100029, PR China

*Corresponding author. E-mail address: liudh@mail.buct.edu.cn (D.L.);

Contents

S1. PXRD Patterns, N_2 Adsorption-Desorption Isotherms, Contents of Cu, Zn, FT-IR Spectrum of Cu-ZIF-8s and Contents of Cu and Zn in Cu-ZIF-8s

S2. Fitting Results of Kinetic Models

S3. Adsorption Capacities of OFX by Different Adsorbents

S4. Fitting Results of Adsorption Isotherms

S5. SEM Images of Cu-ZIF-8-1 after Adsorption

S1. PXRD Patterns, N_2 Adsorption-Desorption Isotherms, FT-IR Spectrum of Cu-ZIF-8s and Contents of Cu and Zn in Cu-ZIF-8s

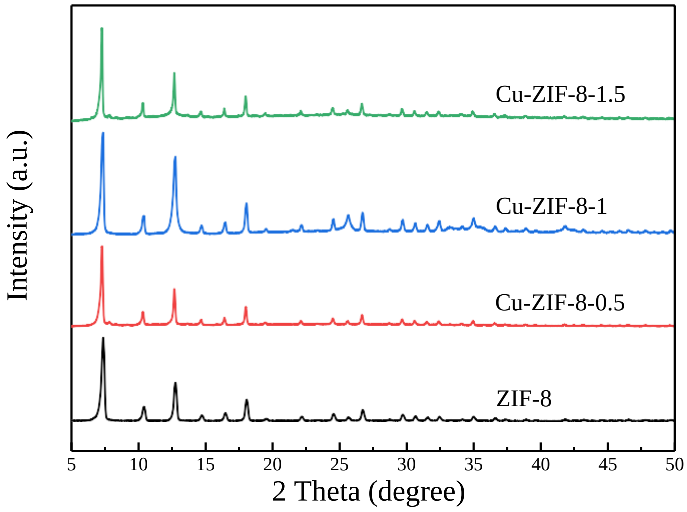

Figure S1. PXRD patterns of ZIF-8, Cu-ZIF-8-0.5, Cu-ZIF-8-1 and Cu-ZIF-8-1.5.

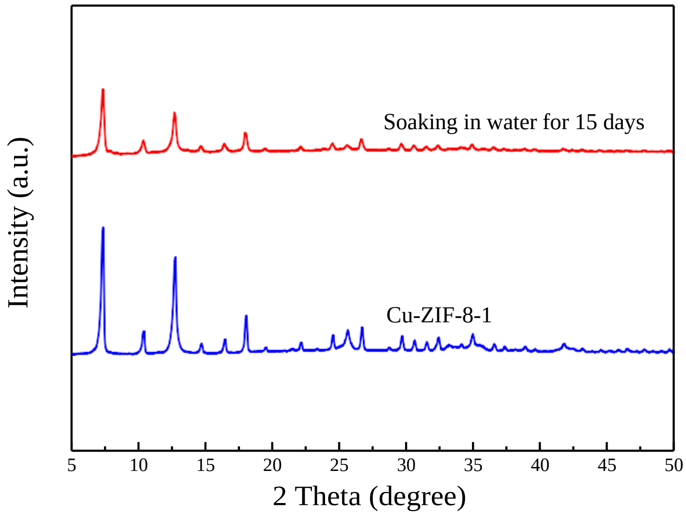

Figure S2. PXRD pattern of Cu-ZIF-8-1 after soaking in water for 15 days.

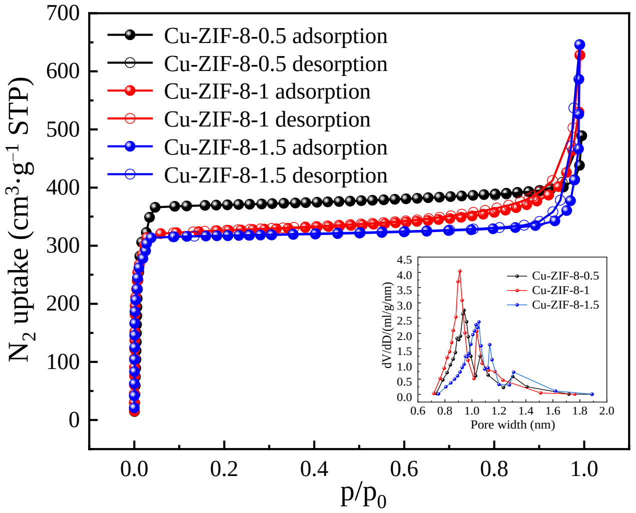

Figure S3. N_2 adsorption-desorption isotherms of Cu-ZIF-8-0.5, Cu-ZIF-8-1 and Cu-ZIF-8-1.5.

Table S1. Specific surface area, mesopore volume, micropore volume and pore size of samples.

MOFs	$S_{Langmuir}$ ($m^2 \cdot g^{-1}$)	V_t ($m^3 \cdot g^{-1}$)	D (nm)
Cu-ZIF-8-0.5	1641.07	0.6837	1.5641
Cu-ZIF-8-1	1438.27	0.7353	1.6124
Cu-ZIF-8-1.5	1388.96	0.7540	1.7239

Table S2. Contents of Cu and Zn in Cu-ZIF-8s.

**MOFs**	**content of Cu (%)**	**content of Zn (%)**	**Cu:Zn**
**Cu-ZIF-8-0.5**	3.633	24.671	1 : 6.79
**Cu-ZIF-8-1**	6.889	20.731	1 : 3.01
**Cu-ZIF-8-1.5**	9.967	17.300	1 : 1.74

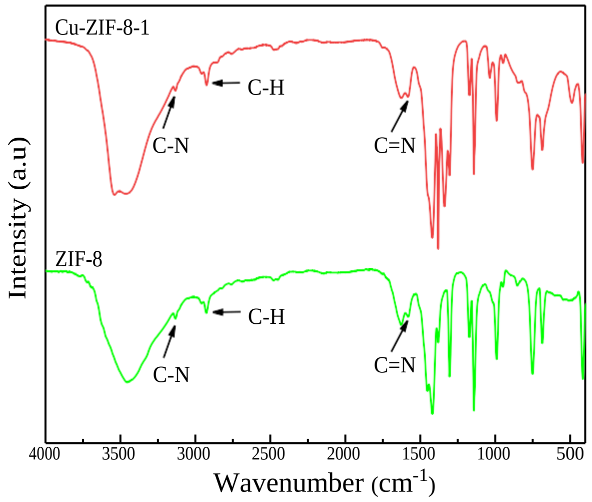

Figure S4. FT-IR spectrum of ZIF-8 and Cu-ZIF-8-1.

S2. Fitting Results of Kinetic Models

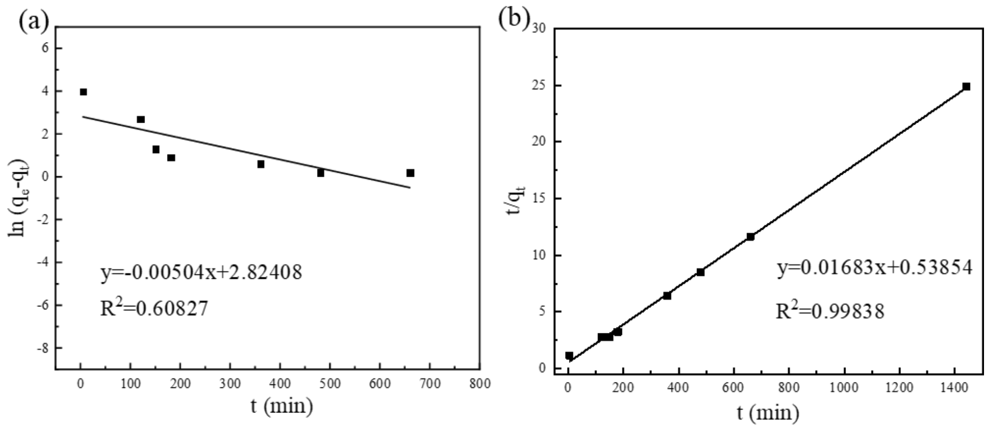

Figure S5. Fitting results of kinetic models on ZIF-8: Pseudo-first-order model (a); Pseudo-second-order model (b).

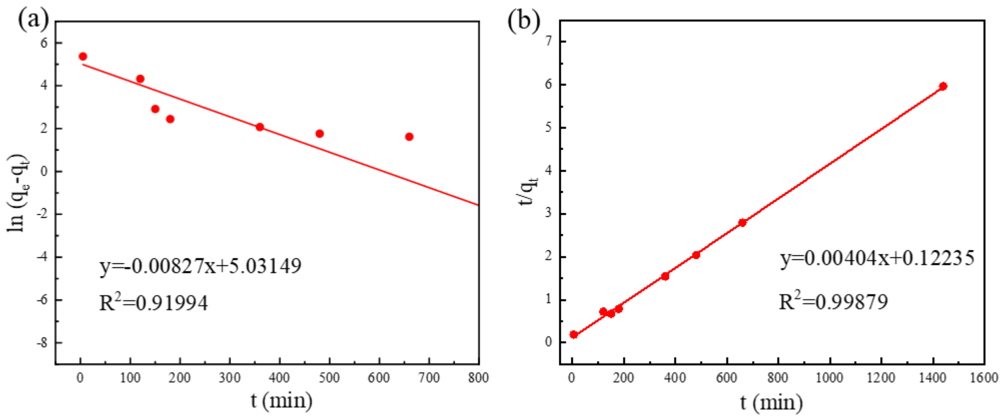

Figure S6. Fitting results of kinetic models on Cu-ZIF-8-1: Pseudo-first-order model (a); Pseudo-second-order model (b).

Table S3. Kinetics model parameters of OFX adsorbed on ZIF-8.

MOF	Pseudo-first-order model			Pseudo-second-order model		
	$q_{e,cal}$ (mg·g^{-1})	k_1 (min^{-1})	R^2	$q_{e,cal}$ (mg·g^{-1})	k_2 (g·min^{-1}·mg^{-1})	R^2
ZIF-8	16.8454	0.0050	0.6083	59.4177	0.0005	0.9984

S3. Adsorption Capacities of OFX by Different Adsorbents

Table S4. Adsorption capacities for the removal of OFX by different adsorbents

Adsorbents	T (°C)	Capacity ($mg \cdot g^{-1}$)	Refs.
Cu-ZIF-8-1	30	599.96	This work
MIL-101(Cr)–SO_3H	30	450.4	[1]
HRP-CsGOn	35	378	[2]
Mesoporous SiO_2	25	290.9	[3]
Activated carbon cloth	25	253	[4]
CVL	55	160.81	[5]
Activated carbon	25	132	[6]
DES-MUiO-66-NH_2	25	97.6	[7]
Co-CNT/NPC 3/1	Room temperature	78.42	[8]
BNNSs	25	72.5	[9]
NBC	25	54.054	[10]
Halloysite clay	35	44.7	[11]
rGO-MoS_2	25	37.31	[12]
DES-RHA	30	30.2	[13]
Fe/Zn + H_3PO_4-SBC	25	25.4	[14]
$Zn_3(BTC)_2$	Room temperature	25.3	[15]
nFe	30	12.8	[16]
RHA	30	7.2	[17]
Met-GO/SA	35	3.436	[18]
GO/SA	35	1.798	[18]

S4. Fitting Results of Adsorption Isotherms

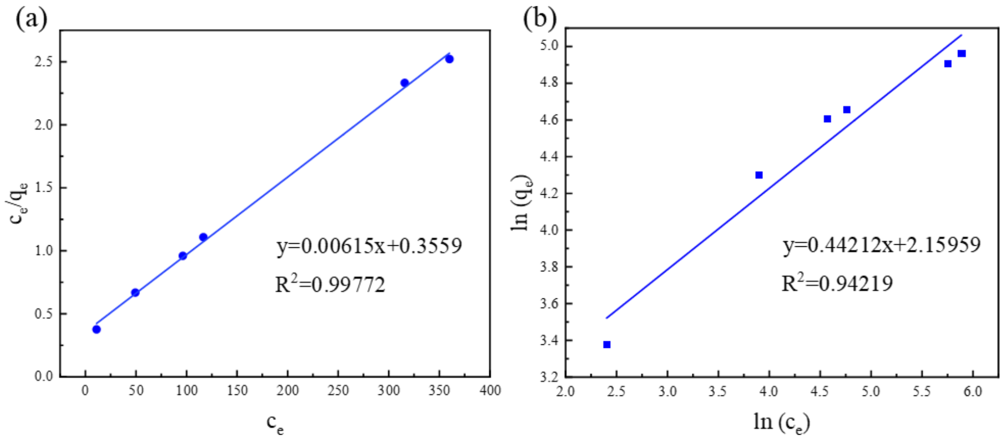

Figure S7. Fitting results of adsorption isotherms on ZIF-8: Langmuir model (a); Freundlich model (b).

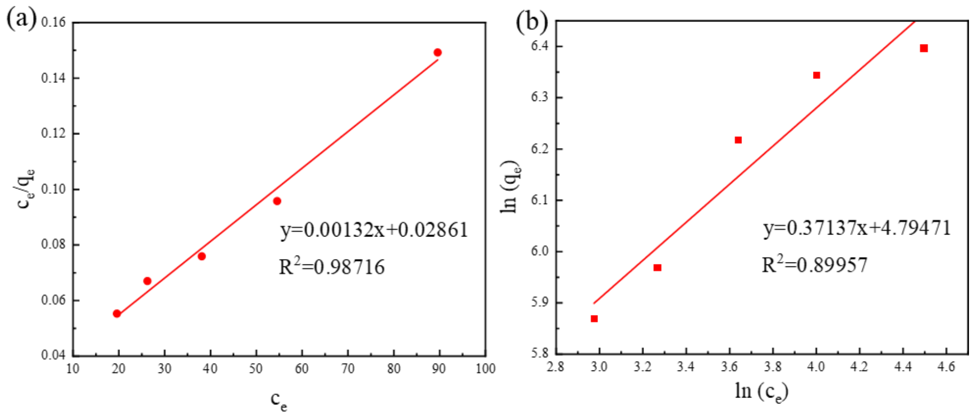

Figure S8. Fitting results of adsorption isotherms on Cu-ZIF-8-1: Langmuir model (a); Freundlich model (b).

Table S5. Model parameters for OFX adsorbed on the ZIF-8.

MOF	Langmuir isotherm model			Freundlich isotherm model		
	q_m ($mg \cdot g^{-1}$)	K_L ($L \cdot g^{-1}$)	R^2	K_F ($(L \cdot mg^{-1})^{1/n} mg \cdot g^{-1}$)	$1/n$ ($g \cdot min^{-1} \cdot mg^{-1}$)	R^2
ZIF-8	162.6016	0.0173	0.9977	8.6676	0.4421	0.9422

S5. SEM Images of Cu-ZIF-8-1 after Adsorption

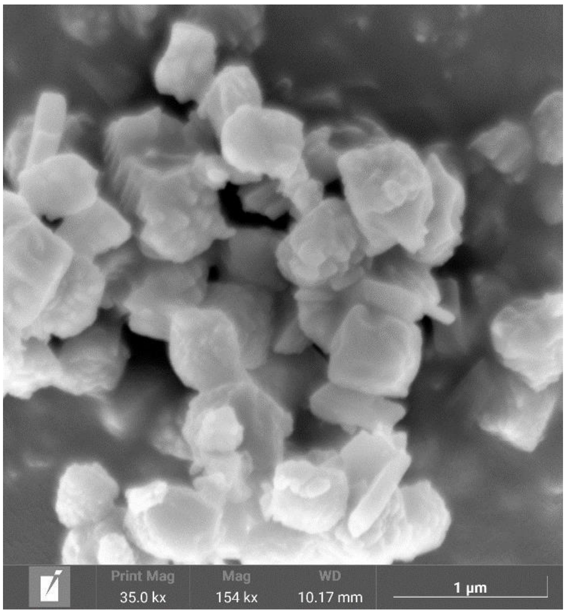

Figure S9. SEM images of Cu-ZIF-8-1 after adsorption (c_0 = 200 mg·L^{-1}).

References

1. Guo, X.; Kang, C.; Huang, H.; Chang, Y.; Zhong, C. Exploration of functional MOFs for efficient removal of fluoroquinolone antibiotics from water. *Micropor. Mesopor. Mater.* **2019**, 286, 84-91, doi:10.1016/j.micromeso.2019.05.025.

2. Suri, A.; Khandegar, V.; Kaur, P.J. Ofloxacin exclusion using novel HRP immobilized chitosan cross-link with graphene-oxide nanocomposite. *Groundw. Sustain. Dev.* **2021**, 12, 100515, doi:10.1016/j.gsd.2020.100515.

3. Goyne, K.W.; Chorover, J.; Kubicki, J.D.; Zimmerman, A.R.; Brantley, S.L. Sorption of the antibiotic ofloxacin to mesoporous and nonporous alumina and silica. *J. Colloid Interface Sci.* **2005**, 283, 160-170, doi:10.1016/j.jcis.2004.08.150.

4. Delpeux-Ouldriane, S.; Gineys, M.; Cohaut, N.; Béguin, F.; Masson, S.; Reinert, L.; Duclaux, L. Adsorption and Desorption of Emerging Water Contaminants on Activated Carbon Fabrics. *Int. J. Environ. Pollut Remediat.* **2016**, 4, 1-6, doi:10.11159/ijepr.2016.001.

5. Antonelli, R.; Martins, F.R.; Malpass, G.R.P.; da Silva, M.G.C.; Vieira, M.G.A. Ofloxacin adsorption by calcined Verde-lodo bentonite clay: Batch and fixed bed system evaluation. *J. Mol. Liq.* **2020**, 315, 113718, doi:10.1016/j.molliq.2020.113718.

6. Kong, Q.; He, X.; Shu, L.; Miao, M.-s. Ofloxacin adsorption by activated carbon derived from luffa sponge: Kinetic, isotherm, and thermodynamic analyses. *Process Saf. Environ. Prot.* **2017**, 112, 254-264, doi:10.1016/j.psep.2017.05.011.

7. Wei X, Wang Y, Chen J, et al. Adsorption of pharmaceuticals and personal care products by deep eutectic solvents-regulated magnetic metal-organic framework adsorbents: Performance and mechanism. *Chem. Eng. J.* **2020**, 392, 124808. doi:10.1016/j.cej.2020.124808.

8. Liang, Y.; Zhang, Q.; Li, S.; Fei, J.; Zhou, J.; Shan, S.; Li, Z.; Li, H.; Chen, S. Highly efficient removal of

quinolones by using the easily reusable MOF derived-carbon. *J. Hazard. Mater.* **2022**, 423, 127181, doi:10.1016/j.jhazmat.2021.127181.

9. Bangari, R.S.; Sinha, N. Adsorption of tetracycline, ofloxacin and cephalexin antibiotics on boron nitride nanosheets from aqueous solution. *J. Mol. Liq.* **2019**, 111376, 293, doi:10.1016/j.molliq.2019.111376.
10. Tang, Y.; Li, Y.; Zhan, L.; Wu, D.; Zhang, S.; Pang, R.; Xie, B. Removal of emerging contaminants (bisphenol A and antibiotics) from kitchen wastewater by alkali-modified biochar. *Sci. Total Environ.* **2022**, 805, 150158, doi:10.1016/j.scitotenv.2021.150158.
11. Wang, Q.; Zhang, J.; Zheng, Y.; Wang, A. Adsorption and release of ofloxacin from acid- and heat-treated halloysite. *Colloids Surf. B Biointerfaces.* **2014**, 113, 51-58, doi:10.1016/j.colsurfb.2013.08.036.
12. Jaswal, A.; Kaur, M.; Singh, S.; Kansal, S.K.; Umar, A.; Garoufalis, C.S.; Baskoutas, S. Adsorptive removal of antibiotic ofloxacin in aqueous phase using rGO-MoS2 heterostructure. *J. Hazard. Mater.* **2021**, 417, 125982, doi:10.1016/j.jhazmat.2021.125982.
13. Kaur, G.; Singh, N.; Rajor, A.; Kushwaha, J.P. Deep eutectic solvent functionalized rice husk ash for effective adsorption of ofloxacin from aqueous environment. *J. Contam. Hydrol.* **2021**, 242, 103847, doi:10.1016/j.jconhyd.2021.103847.
14. Ma, Y.; Li, P.; Yang, L.; Wu, L.; He, L.; Gao, F.; Qi, X.; Zhang, Z. Iron/zinc and phosphoric acid modified sludge biochar as an efficient adsorbent for fluoroquinolones antibiotics removal. *Ecotox. Environ. Saf.* **2020**, 196, 110550, doi:10.1016/j.ecoenv.2020.110550.
15. Capsoni, D.; Guerra, G.; Puscalau, C.; Maraschi, F.; Bruni, G.; Monteforte, F.; Profumo, A.; Sturini, M. Zinc Based Metal-Organic Frameworks as Ofloxacin Adsorbents in Polluted Waters: ZIF-8 vs. Zn3(BTC)2. *Int. J. Environ. Res. Public Health* **2021**, 18, 1433, doi:10.3390/ijerph18041433.
16. Weng X, Cai W, Owens G, et al. Magnetic iron nanoparticles calcined from biosynthesis for fluoroquinolone antibiotic removal from wastewater. *J. Clean. Prod.* **2021**, 319: 128734. doi:10.1016/j.jclepro.2021.128734.

17. Kaur, G.; Singh, N.; Rajor, A. Ofloxacin adsorptive interaction with rice husk ash: Parametric and exhausted adsorbent disposability study. *J. Contam. Hydrol.* **2021**, 236, 103737, doi:10.1016/j.jconhyd.2020.103737.

18. Yadav, S.; Asthana, A.; Singh, A.K.; Chakraborty, R.; Vidya, S.S.; Singh, A.; Carabineiro, S.A.C. Methionine-Functionalized Graphene Oxide/Sodium Alginate Bio-Polymer Nanocomposite Hydrogel Beads: Synthesis, Isotherm and Kinetic Studies for an Adsorptive Removal of Fluoroquinolone Antibiotics. *Nanomaterials-Basel* **2021**, 11, 568, doi:10.3390/nano11030568.
